# Glucagon-like Peptide-1 Receptor Agonists and Diabetic Osteopathy: Another Positive Effect of Incretines? A 12 Months Longitudinal Study

**DOI:** 10.1007/s00223-024-01240-1

**Published:** 2024-06-12

**Authors:** Antonella Al Refaie, Leonardo Baldassini, Caterina Mondillo, Elena Ceccarelli, Roberto Tarquini, Luigi Gennari, Stefano Gonnelli, Carla Caffarelli

**Affiliations:** 1 Section of Internal Medicine, Department of Medicine, Surgery and Neuroscience, University of Siena, Policlinico Le Scotte, Viale Bracci 2, 53100 Siena, Italy; 2 Division of Internal Medicine I, San Giuseppe Hospital, 50053 Empoli, Italy

**Keywords:** Type 2 diabetes mellitus (T2DM), Bone mineral density (BMD), Dual-energy X-ray absorptiometry (DXA), Radiofrequency echographic multispectrometry (REMS), Trabecular bone score (TBS), Bone turnover markers, Dulaglutide, Semaglutide

## Abstract

Diabetic osteopathy is a frequent complication in patients with type 2 diabetes mellitus (T2DM). The association between T2DM and increased fracture risk has led to study the impact of new antidiabetic drugs on bone metabolism. Glucagon-like peptide-1 receptor agonists (GLP-1RAs) are incretin mimetic drugs which have many pleiotropic properties. The relationship between GLP-1RAs and bone is very complex: while in vitro and animal studies have demonstrated a protective effect on bone, human studies are scarce. We led a 12 months longitudinal study evaluating bone changes in 65 patients withT2DM for whom a therapy with GLP-1RAs had been planned. Fifty-four T2DM patients completed the 12-month study period; of them, 30 had been treated with weekly dulaglutide and 24 with weekly semaglutide. One-year therapy with GLP-1RAs resulted in a significant reduction in weight and BMI. Bone mineral density (BMD), bone metabolism, trabecular bone score (TBS), adiponectin, and myostatin were evaluated before and after 12 months of GLP-1RAs therapy. After 12 months of therapy bone turnover markers and adiponectin showed a significant increase, while myostatin values showed a modest but significant reduction. BMD-LS by DXA presented a significant reduction while the reduction in BMD-LS by REMS was not significant and TBS values showed a marginal increase. Both DXA and REMS techniques showed a modest but significant reduction in femoral BMD. In conclusion, the use of GLP-1RAs for 12 months preserves bone quality and reactivates bone turnover. Further studies are needed to confirm whether GLP-1RAs could represent a useful therapeutic option for patients with T2DM and osteoporosis.

**Supplementary Information:**

The online version contains supplementary material available at 10.1007/s00223-024-01240-1.

## Introduction

Diabetes mellitus (DM), the most common chronic metabolic disorder worldwide, leads to many different microvascular and macrovascular complications; today even bone is recognized as a target of diabetes, in fact both type 1 DM (T1DM) and type 2 DM (T2DM) patients have increased risk of fractures compared to normal subjects [1]. If bone fragility in T1DM can be partially explained by reduced lumbar and femoral bone mineral density (BMD), in T2DM subjects BMD is normal or even increased compared to healthy subjects. This paradox can find an explanation in several factors, including reduced bone turnover and strength, worse bone quality, alterations in bone microarchitecture and in composition of bone matrix [1].

The association between diabetes mellitus and increased fracture risk has led to study the impact of antidiabetic drugs on bone metabolism. It’s known that thiazolidinediones reduce bone density; insulin increases the risk of fractures (probably as a result of an increase in falls, related to episodes of hypoglycemia and the fact that insulin is used in the most serious cases); metformin has neutral effects on bone, instead studies are not unique for sodium/glucose cotransporter 2 (SGLT-2) (in particular canagliflozin) [2–4]. Incretins are hormones produced in the gastrointestinal tract, the main ones are: glucagon-like peptide 1 (GLP-1) produced by L cells and gastric inhibitory peptide (GIP) produced by K cells. These hormones are secreted after meals, especially GLP-1 and have a role in glycemic control; in particular, incretins increase insulin secretion, reduce glucagon secretion, slow down motility and gastric emptying, reduce appetite and improve insulin sensitivity [5]. Glucagon-like peptide-1 receptor agonists (GLP-1RAs) are incretin mimetic drugs; they are a relatively new class of drugs that are revolutionizing T2DM therapy. In fact, in addition to their hypoglycemic action, GLP-1RAs appear to have positive effects on weight, cardiovascular risk, and other additional pleiotropic properties. All these reasons make these drugs very attractive for diabetic patients. Therefore, in the most recent guidelines, GLP-1RAs are considered first-line drugs for the therapy of T2DM patients [6].

There are several GLP-1RAs available, each with varying degrees of similarity to native GLP-1 and different potencies in reducing HbA1c levels [7]. The relationship between GLP-1RAs and bone is very complex. Studies conducted in vitro and on animals have shown that GLP-1RAs, and in particular liraglutide and exenatide, appear to have a positive effect on bone by stimulating new bone formation and reducing bone resorption. In fact, many of these studies have shown that treatment with GLP-1RAs reduces carboxy-terminal telopeptide (CTX) levels and tends to increase those of alkaline phosphatase (ALP) and amino-terminal propeptide of type I pro-collagen (PINP) [8, 9]. Furthermore, some studies conducted on ovariectomized mice or rats have reported that the administration of GLP-1RAs increases BMD and appears to have a protective effect on bone microstructure [10, 11].

However, although in vitro and animal studies have overall demonstrated a positive effect of GLP-1RAs on bone turnover and BMD, to date, human studies evaluating the effects of GLP-1RAs on bone metabolism, BMD, and fragility fractures are scarce and have reported quite conflicting results [9].

The aim of this observational study was to evaluate in a cohort of adult and elderly patients with T2DM the effects on bone turnover and bone status of 1-year treatment with the GLP-1RAs.

## Patients and Methods

### Patients

For this study, we enrolled patients of both sexes affected by T2DM and referred to the Diabetology and Metabolic Diseases Unit of the Department of Internal Medicine at the University Hospital of Siena (Italy) for whom the diabetologist had planned to start treatment with GLP-1RAs. In the time period between May 2022 and December 2022 80 consecutive T2DM patients initiated therapy with GLP-1RAs (dulaglutide or semaglutide) and were evaluated for the study. On the basis of inclusion/exclusion criteria, 65 T2DM patients (30 men and 35 women) were included in this 12-month study; of them 36 had received a prescription for dulaglutide and 29 for semaglutide. The inclusion criteria were as follows: patients who started for the first time therapy with GLP-1RAs, age between 50 and 80 years and HbA1c > 6.5% and < 10%. Patients with T2DM who had already undertaken therapy with GLP-1RAs or patients who were receiving therapy with insulin, thiazolodinediones, SGLT2 inhibitors, acarbose were excluded. Patients previously treated with antiosteoporosis drugs and those who were suffering illness (multiple myeloma, hyperparathyroidism, osteogenesis imperfecta, cancer, osteometabolic diseases) or were receiving therapies able to influence bone metabolism were excluded. The maintenance dose for dulaglutide (1.5–3.0 mg weekly) and semaglutide (1.0–2.0 mg weekly) was reached after a dose escalation period of 8–12 weeks as indicated in the leaflet.

**Fig. 1:**
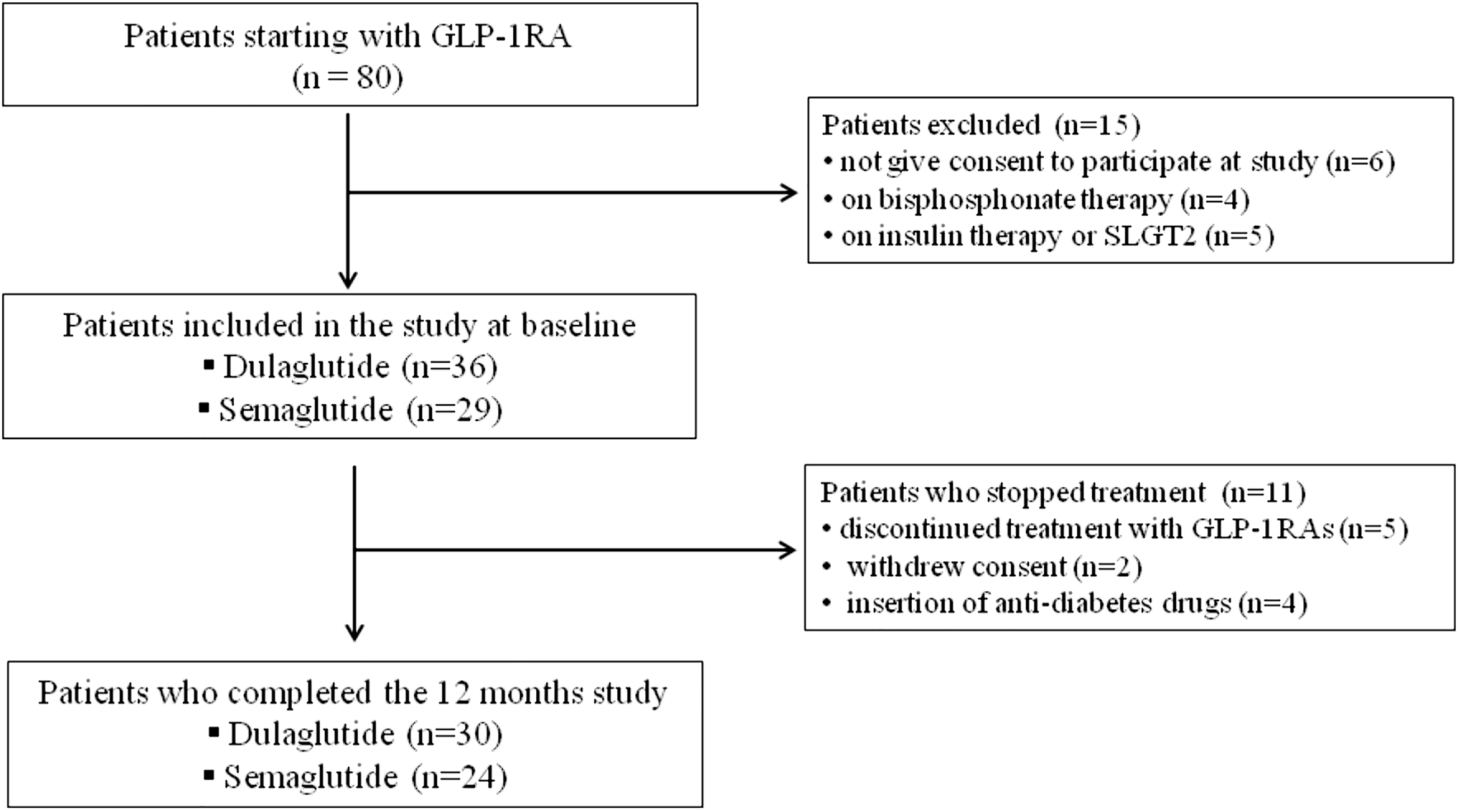
The study flow-chart

The flow chart of the T2DM patients involved in the study is shown in Fig. 1. An informed written consent was obtained from all participants, and the research protocol received approval from the Institutional Review Board of Siena University Hospital (ID-21211/21). Prior to statistical analysis, all collected data underwent anonymization procedure.

## Methods

All patients underwent all required clinical, laboratory, and instrumental evaluations before starting the therapy and after 12 months. In particular, a detailed medical history was collected and all patients underwent measurement of weight and height; moreover, body mass index (BMI) which expresses the ratio between weight in kilograms divided by the square of height in meters was calculated.

After fasting for at least 12 h, patients underwent blood sampling for the evaluation of: glycemia, glycated hemoglobin, creatinine, calcium, phosphate, C-terminal telopeptide of type 1 collagen (CTX), bone isoenzyme of alkaline phosphatase (B-ALP), parathyroid hormone (PTH), 25hydroxyvitaminD (25OHD), adiponectin, sclerostin, and myostatin. The measurement was carried out with a colorimetric method (Autoanalyzer, Falcor 350 Menarini). Serum CTX was measured by an ELISA method (Immunotopics INT, San Clemente, CA, USA) and the intra- and inter-assay precision was 2.5 and 3.5%, respectively. Serum B-ALP was measured by a chemiluminescence immunoassay method (LIAISON BAP Ostase, DiaSorin Inc., Stillwater, MN, USA); in our institution, the intra-and inter-assay coefficients of variation for B-ALP were 4.2% and 7.9%, respectively. Serum 25OHD was determined by a chemi- luminescence immunoassay (LIAISON 25OHD Total Assay, DiaSorin Inc, Stillwater, MN, USA); in our institution, the intra- and inter-assay coefficients of variation were 6.8% and 9.2%, respectively. Serum PTH was assessed by immunoradiometric assay (Total Intact PTH, Antibodies Lab. Inc.; Santee, CA, USA) and the intra- and inter-assay coefficients of variation were 3.6% and 4.9%, respectively. Adiponectin was measured by a commercially available radioimmunoassay (adiponectin human RIA kit; DRG International, Mountainside, NJ), which measures multiple forms of adiponectin (trimer, hexamer, and high-molecular weight forms). The results were expressed in micrograms per milliliter; the intra- and interassay coefficients of variation were 3.8% and 8.4%, respectively, at concentrations between 3 and 15 microg/mL. Serum myostatin was determined by an ELISA method (Human Myostatin, Elisa Kit, My BioSource, San Diego, CA,). In our institution the intra- and inter-assay coefficients of variation were 5% and 8%, respectively**.** Serum sclerostin levels were assessed using a quantitative sandwich ELISA from Biomedica (Biomedica Gruppe, Vienna, Austria**),** with intra- and inter-assay CV of 4% and 5.5%, respectively.

In all subjects we measured BMD at the lumbar spine (LS-BMD), at femoral neck (FN-BMD) and total hip (TH- BMD) using a dual-energy X-ray absorptiometry device (Discovery W, Hologic, Waltham, MA, USA). All DXA scans were performed according to the standard clinical routine procedures. Osteoporosis and osteopenia were diagnosed according to the World Health Organization (WHO) definition: a T value lower than − 2.5 was diagnosed as osteoporosis and a T value less than − 1.0 but higher than − 2.5 was diagnosed as osteopenia; sex-matched Italian reference data were used for the calculation of T-score. For a better estimate of bone tissue microarchitecture, we also calculated the Trabecular Bone Score (TBS). TBS was calculated by using TBS iNsight software (Version 2.1, Medimaps SA, Bordeaux, France) in an operator-independent automated manner. The Trabecular Bone Score was calculated from the standard DXA scan of the antero-posterior lumbar spine.

BMD was also measured by Radiofrequency Echographic Multispectrometry (REMS) technology. REMS scans were performed employing an echographic device (EchoStation, Echolight Spa, Lecce, Italy), using a convex transducer operating at the nominal frequency of 3.5 MHz [12]. During REMS examination, the probe is placed on the abdomen or on the hip in order to visualize the target bone, the operator has to set the appropriate values of scan depth and transducer focus. The selected measured data are finally synthesized in a patient specific spectrum of the considered bone target, which undergoes an advanced comparison with gender, age-, site, and BMI matched reference spectral models extracted from a dedicated database [12]. Some papers have reported that REMS technique presents a good precision and a diagnostic accuracy similar [13] or, at least in patients with T2DM, superior to DXA [14].

### Statistical Analysis

All values were expressed as mean ± SD. Clinical data and initial values of the measured variables in the study groups were compared using Student’s t-test for unpaired data. For DXA, REMS, and TBS parameters, the absolute changes over time for each T2DM subjects were expressed as a percentage of the baseline values. Two-tailed paired t-tests and Wilcoxon matched-pairs signed-ranks tests were used, when appropriate, to compare the changes at each time point with the baseline values. Two-tailed Student’s t-test and Mann–Whitney U-test were used to compare the difference between patient groups. A p-value < 0.05 was considered statistically significant. All tests were performed using the SPSS statistical package for Windows version 16.0 (SPSS Inc., Chicago).

## Results

**Fig. 2:**
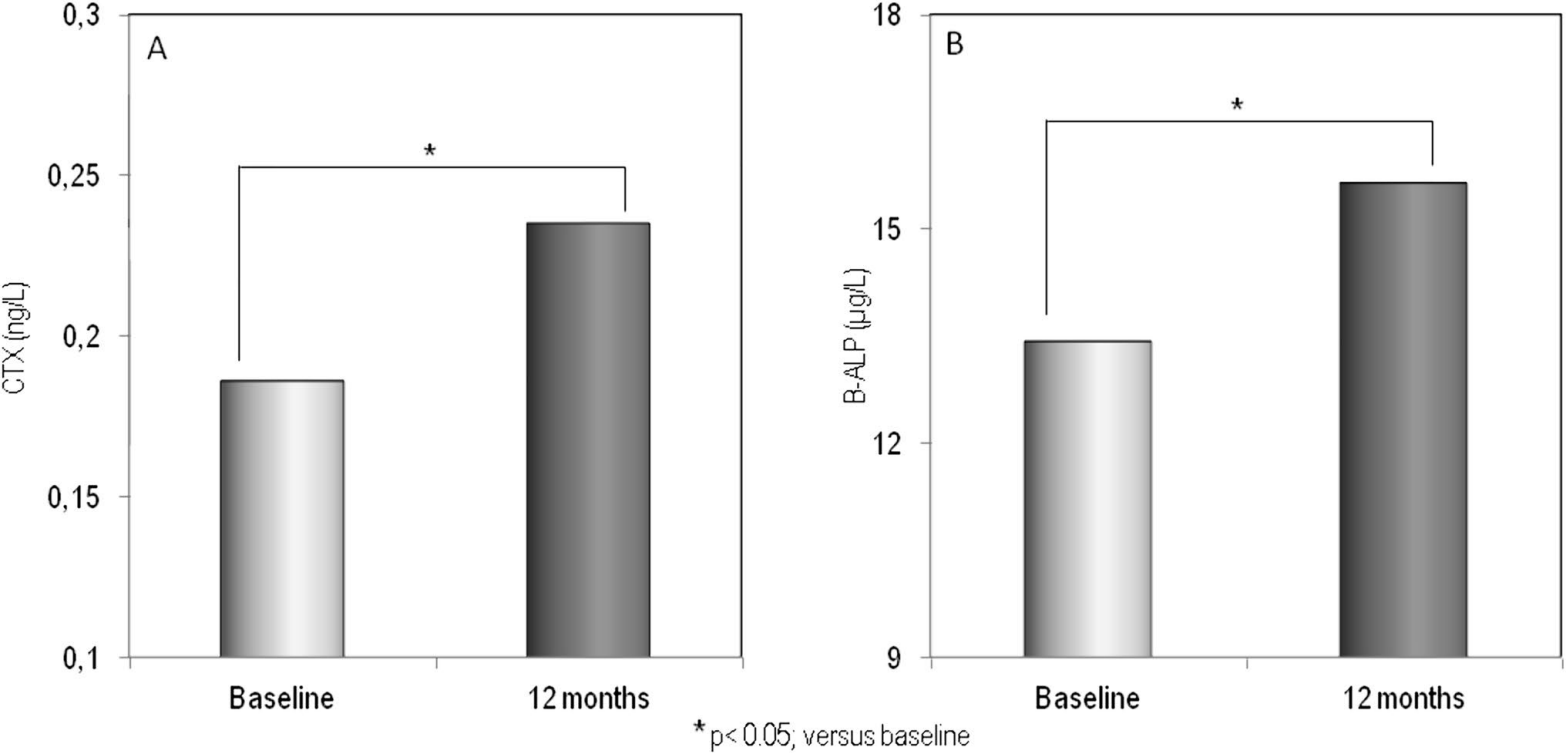
Serum levels of CTX (**A**) and B-ALP (**B**) in T2DM patients at baseline and after 12 months of therapy with GLP-1RAs

**Fig. 3:**
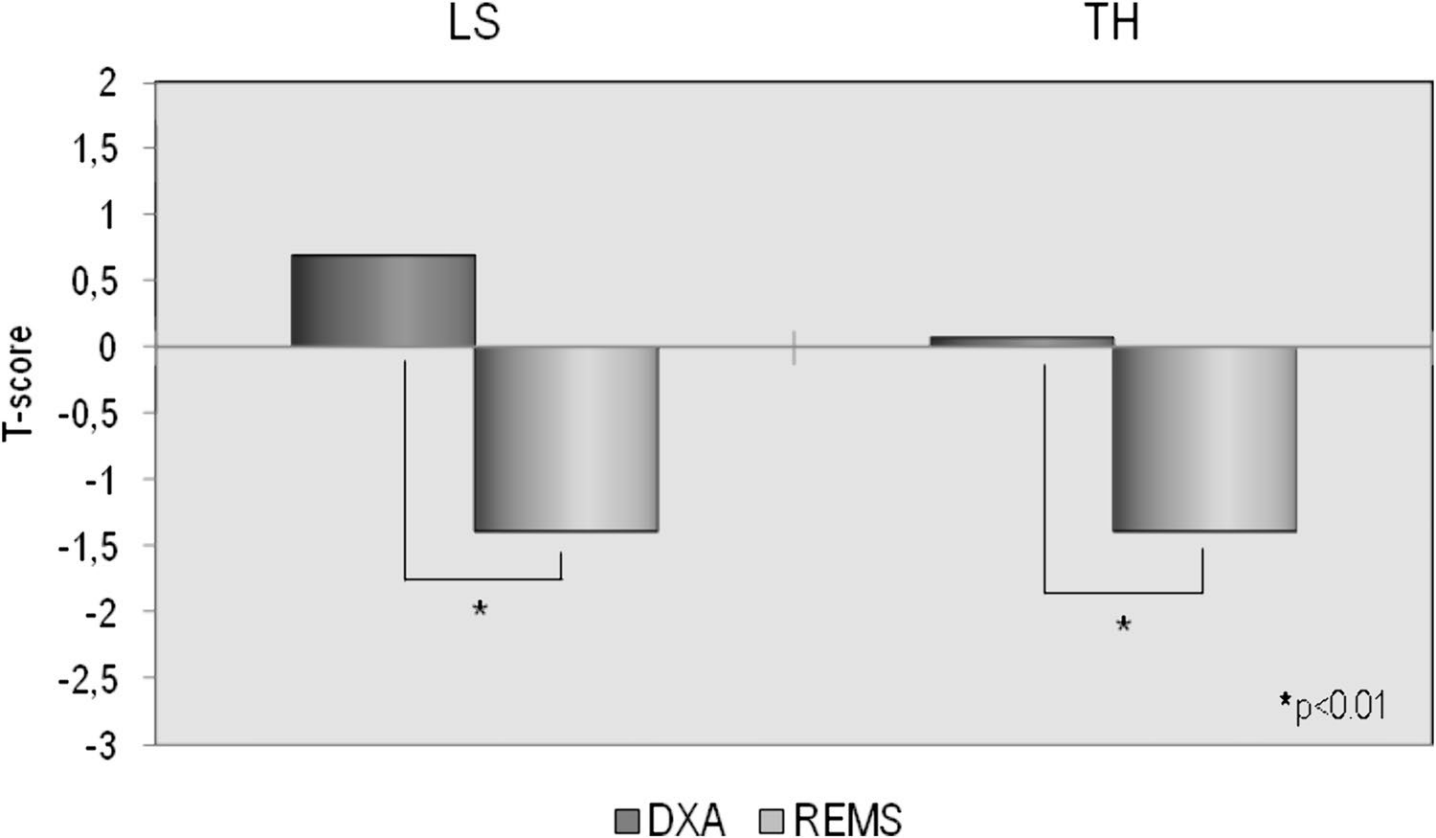
Values of BMD expressed as T-score at lumbar spine (LS) and at total hip (TH) by DXA and REMS technique in T2DM patients in therapy with GLP-1RAs

**Fig. 4:**
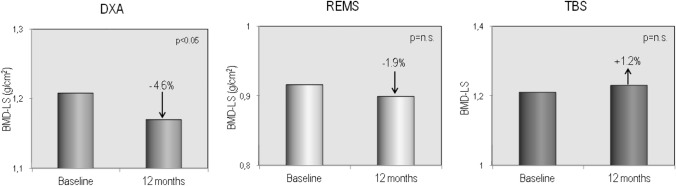
Mean percent changes from baseline in BMD at lumbar spine by DXA, by REMS and in TBS in T2DM patients at baseline and after 12 months of therapy with GLP-1RAs

**Fig. 5:**
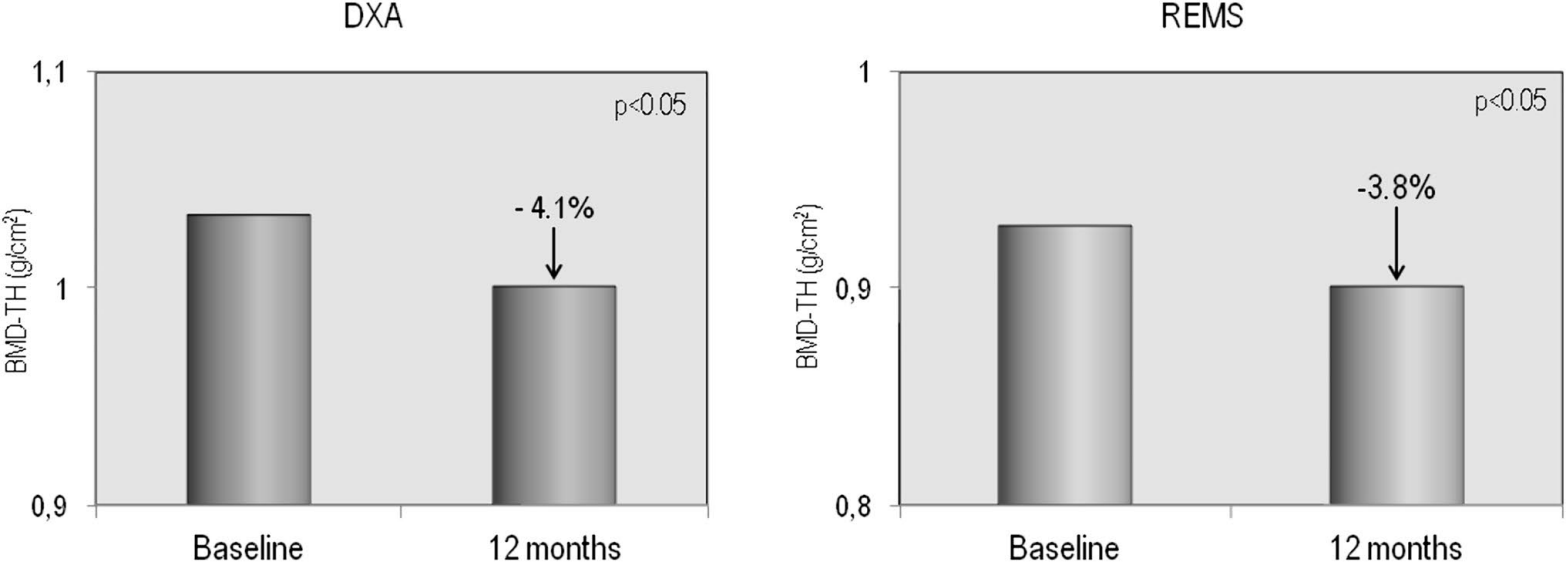
Mean percent changes from baseline in BMD at total hip by DXA and by REMS in T2DM patients at baseline and after 12 months of therapy with GLP-1RAs

**Fig. 6:**
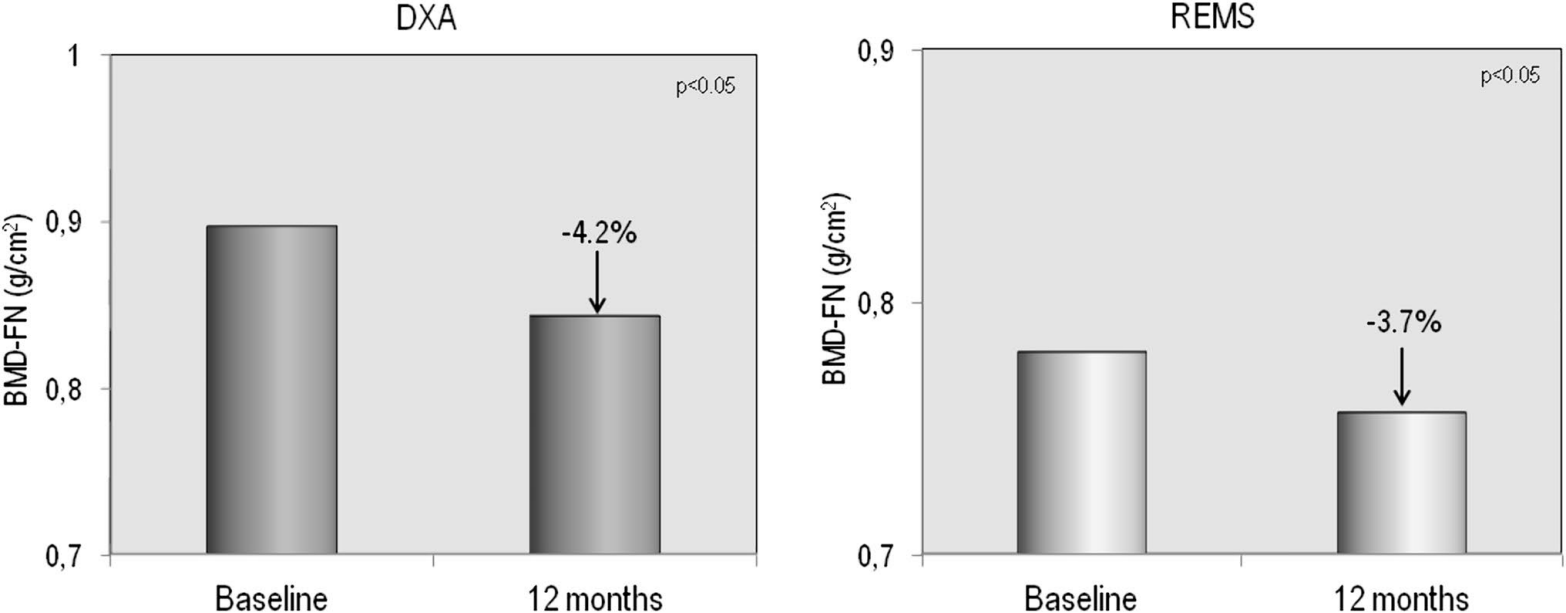
Mean percent changes from baseline in BMD at femoral neck by DXA and by REMS in T2DM patients at baseline and after 12 months of therapy with GLP-1RAs

The demographic, clinical, and laboratory characteristics of the study population at baseline and after 12 months of therapy with GLP-1RAs are reported in Table 1. Fifty-four T2DM patients (26 males and 28 females) completed the 12-month study period; of them, 30 had been treated with dulaglutide and 24 with semaglutide. As expected, 1 year of therapy with GLP-1RAs resulted in a significant reduction in glycated hemoglobin and, above all, a significant weight loss ( − 4.2 kg) and a significant reduction in BMI (from 30.2 to 28.6 kg/m2). Furthermore, bone turnover markers (B-ALP and CTX) and adiponectin showed a significant increase, while myostatin values showed a modest but significant reduction. No significant changes in serum levels of calcium, phosphate, PTH, 25OHD, sclerostin, and creatinine were observed. Figure 2 shows the serum levels of B-ALP and CTX at baseline and after 12 months of therapy with the GLP-1RAs dulaglutide or semaglutide. It is clear that both markers of bone turnover showed a modest but significant increase in their serum levels at the end of the study period. Baseline femoral and vertebral BMD values, expressed as T-score and measured with both DXA and REMS techniques, are shown in Fig. 3. At baseline, the T-score values by REMS were markedly lower than those obtained with DXA and especially at the vertebral level. Figure 4 shows the values of BMD at lumbar spine, assessed by both DXA and REMS techniques, and the values of TBS at baseline and at the end of study period. It is clear that LS-BMD by DXA presented a significant reduction ( − 4.6%) while the reduction in LS-BMD by REMS was smaller and not significant ( − 1.9%); on the contrary, TBS values showed a marginal increase (Fig. 4). Figures 5 and 6 show the values of BMD at total hip (TH-BMD) and at femoral neck (FN-BMD), as assessed by both DXA and REMS techniques, at baseline and the end of study period. Both DXA and REMS techniques showed a significant (*p* < 0.05) reduction in both TH-BMD ( − 4.1% and − 3.8%, respectively) and FN-BMD ( − 4.2% and − 3.7%, respectively). Both at baseline and at the end of the study no significant differences in anthropometric, densitometric, and laboratory parameters were observed between T2DM patients treated with dulaglutide and those treated with semaglutide (Supplementary material).

**Table 1:** Demographic, clinical, and laboratory characteristics of the study population at baseline and after 12 months of therapy with the GLP-1RAs

	Baseline	12 months	*p*
M/F	65 (30/35)	54 (26/28)	
Age (yrs)	66.4 ± 8.45	67.3 ± 8.5	n.s
Weight (Kg)	84.38 ± 12.82	80.19 ± 11.89	0.01
Height (cm)	167.87 ± 8.35	167.33 ± 7.95	n.s
BMI (Kg/m^2^)	30.24 ± 4.52	28.68 ± 3.73	0.01
Glycated hemoglobin (mmol/mol)	60.67 ± 21.00	50.92 ± 8.99	0.05
Calcium (mg/dl)	9.51 ± 0.53	9.48 ± 0.67	n.s
Phosphate (mg/dl)	3.68 ± 0.63	3.91 ± 0.74	n.s
Creatinine (mg/dl)	0.96 ± 0.33	1.00 ± 0.39	n.s
25OHD (ng/ml)	21.07 ± 9.57	22.55 ± 8.11	n.s
PTH (pg/ml)	27.36 ± 23.07	30.55 ± 21.87	n.s
Sclerostin (pmol/L)	53.68 ± 36.73	55.52 ± 37.75	n.s
Adiponectin (µg/ml)	9.46 ± 6.16	11.58 ± 7.05	0.01
Myostatin (ng/ml)	13.02 ± 2.62	11.05 ± 2.12	0.05

## Discussion

An interesting finding of this study is that one year of GLP-1RAs therapy markedly increases bone turnover markers in patients with T2DM. It is known that patients with T2DM, despite a normal or more often increased BMD, present an impaired bone quality which is mainly attributed to a reduced osteoblastic activity documented by reduced levels of markers of new bone formation [15–17]*.* Moreover, a meta-analysis of 66 studies by Hygum et al. showed that both markers of bone formation (osteocalcin) and of bone resorption (CTX) were significantly lower in patients with T2DM than in controls [18]. Numerous in vitro and animal studies have highlighted that incretins stimulate new bone formation. In particular, GLP-1RAs were able to promote upregulation of RUNX2, Beta catenin, alkaline phosphatase, and P1NP that express the maturation and activity of osteoblasts [9, 19]. Some clinical studies conducted in patients with T2DM or overweight treated with liraglutide at doses between 1.2 and 1.8 mg daily have documented a significant increase in P1NP, thus confirming the positive effect on bone formation [21]. Therefore, the results of our study are in agreement with the literature regarding the effect of incretins on markers of bone formation, while they are in apparent contrast for the variations in bone resorption. Indeed, preclinical studies have reported that GLP-1RAs inhibit osteoclastogenesis and reduce serum levels of CTX and urinary deoxypyridinolines [8, 9, 11]. Instead, the two clinical studies conducted in patients with T2DM [21] or obese patients [20] treated with liraglutide reported a marginal and non-significant increase in CTX. However, the increase in both parameters of bone formation and resorption is likely due to the reactivation of bone remodeling, resulting in coupled changes in both processes.

Another finding of this study is that in patients with T2DM one-year therapy with dulaglutide or semaglutide reduces BMD assessed with both DXA and REMS techniques, especially at the level of the femoral site. The results of our study may seem in partial agreement with the study by Cai et al., carried out in T2DM patients treated with dulaglutide (1.5 mg weekly) for 52 weeks, which reported a significant decrease in BMD at femoral level while the BMD at lumbar spine remained unchanged [22]. Moreover, in the study by Hygum et al., treatment with liraglutide for 26 weeks did not cause changes in BMD despite a weight loss [21]. Instead, Huang's study reported that T2DM patients who switched from a dipeptidyl peptidase-4 inhibitors (DPP-4i) to a GLP-1RAs had, in addition to better glycemic control and a marked weight loss, a significant reduction in BMD [23]. Intervention studies, including randomized controlled trials and meta-analyses, indicate that non-surgical weight reduction achieved through caloric restriction, either alone or coupled with low-impact aerobic exercise, leads to elevated levels of bone turnover markers and reduced bone mineral density values [24]**.** Although there are studies showing a decrease in BMD in hip and spine, a large meta-analysis showed that diet-induced weight loss affected the BMD at the femoral sites but not at lumbar spine [24]. Some authors attributed the more pronounced decline of hip BMD compared to the lumbar spine to the presence of artifacts (such as aortic calcifications, osteophytes; osteoarthritis) that may reduce the accuracy of BMD evaluation [24]. However, the fact that in this study the lumbar BMD measured with the REMS technique, which is able to exclude artifacts, presented a similar reduction to the BMD by DXA could exclude this hypothesis [14]. It appears more likely that this discrepancy can be ascribed to a greater sensitivity of femoral BMD to changes in weight, which is related to the different distribution of trabecular and cortical bone in the two skeletal sites [25]. Moreover, recent meta-analyses have not identified any notable difference in fracture risk between GLP-1RAs and either placebo or alternative antidiabetic medications [9, 25, 26]. Therefore, the slight loss of BMD observed by both DXA and REMS would reflect weight loss rather than an actual negative effect on BMD. On the other hand, fracture risk in T2DM subjects does not depend only on BMD but it is a more complex concept concerning microarchitecture, trabecular bone, strength, and resistance. It’s for this reason that trabecular bone score (TBS) appears to be more accurate than BMD in diabetic osteopathy to predict fracture risk; in fact several studies have confirmed that TBS is a valid index of bone microarchitecture in T2DM patients [27]. It is interesting to observe how in our study TBS did not change after the 12-month treatment with GLP-1RAs. These findings support the thesis that GLP-1RAs have no negative effects on bone quality. This observation is also supported by the fact that in the Hygun’s study, carried out in a population similar to ours, the bone structure assessed with the HRpQCT scanner remained unchanged despite the significant weight loss induced by liraglutide [21]. Unfortunately, this latter study lasted only six months and may not be enough time to show any changes in bone quality. Also the increase in sclerostin observed in this study is presumably due to weight loss [28].

Another interesting finding of this study is represented by the significant increase in adiponectin at the end of 12 months of treatment with GLP-1RAs which could result in greater protection at bone level. In fact, both in vivo and in vitro studies, have reported that adiponectin deficiency would trigger an increase in osteoclastogenesis, adipogenesis and a reduction in osteogenesis [29]**.** This data confirms a recent meta-analysis that evaluated the effects of GLP-1RAs on adiponectin and found that GLP-1RAs were able to increase adiponectin levels [30]. Although molecular mechanisms underlying the elevation in circulating adiponectin concentrations following GLP1-RAs are yet to be elucidated, higher values of adiponectin may reduce inflammatory cytokines and oxidative stress with positive effects on bone metabolism [30]. However, the relationships between adiponectin levels and BMD values in humans are complex and requires further studies. Indeed some clinical studies have found an inverse correlation between adiponectin levels and BMD [31, 32], while a longitudinal study conducted in the Swedish MrOs cohort, observed that the risk of fracture increased in parallel with the rise in serum adiponectin levels [33]. Myostatin is a cytokine which negatively regulates the growth and development of muscle cells. Recent research has shown that myostatin might have an essential role in bone metabolism too; in particular, overexpression of myostatin promoted osteoclastogenesis [34]. The reduction of myostatin after 12 months of therapy, would be another result supporting the possible positive effect of GLP-1RAs on bone.

Our study presents some limitations. First, the lack of a control group including T2DM patients undergoing antidiabetic drugs with proven absence of relevant effects on bone. Second, the absence of a control group undergoing weight loss solely through lifestyle changes does not allow us to determine whether the changes in both bone markers and BMD are attributable only to weight loss or to direct effect of GLP-1RAs. Third, in this study, leptin, an adipokine that has been reported to have effects on bone, was not measured [35, 36]. Fourth, the 12-month study period is too short to confidently evaluate a drug's effects on BMD. But this study also has some strengths, in particular, to our knowledge, this is one of the first studies lasting 12 months that has evaluated the effects of GLP-1RAs on BMD in T2DM patients and it is certainly the first study to have also evaluated BMD by REMS, TBS, adiponectin, and myostatin.

## Conclusion

This study has demonstrated that a 1-year treatment with GLP-1RAs is capable of reactivating bone turnover and preserving bone quality of the spine in patients with T2DM. The modest reduction in BMD, especially at the femoral level, that we observed after 12 months of therapy with the GLP-1RAs dulaglutide and semaglutide is probably due to the significant weight loss and does not seem to interfere with bone quality. Further studies are needed to confirm whether GLP-1RAs could represent a useful therapeutic option for patients with T2DM and osteoporosis.

### Supplementary Information

Below is the link to the electronic supplementary material.

Supplementary file1 (DOCX 17 KB)

## Data Availability

Data will be available upon reasonable request.

## References

[1] de Liefde II, van der Klift M, de Laet CE, van Daele PL, Hofman A, Pols HA (2005). Bone mineral density and fracture risk in type-2 diabetes mellitus: the Rotterdam Study. Osteoporos Int.

[2] Betteridge DJ (2011). Thiazolidinediones and fracture risk in patients with Type 2 diabetes. Diabet Med.

[3] Hu J, Han J, Jin M, Jin J, Zhu J (2023). Effects of metformin on bone mineral density and bone turnover markers: a systematic review and meta-analysis. BMJ Open.

[4] Blau JE, Taylor SI (2018). Adverse effects of SGLT2 inhibitors on bone health. Nat Rev Nephrol.

[5] Nauck MA, Meier JJ (2018). Incretin hormones: their role in health and disease. Diabetes Obes Metab.

[6] ElSayed NA, Aleppo G, Aroda VR, Bannuru RR, Brown FM, Bruemmer D, Collins BS, Hilliard ME, Isaacs D, Johnson EL, Kahan S, Khunti K, Leon J, Lyons SK, Perry ML, Prahalad P, Pratley RE, Seley JJ, Stanton RC, Gabbay RA, on behalf of the American Diabetes Association. 9 (2023). Pharmacologic approaches to glycemic treatment: standards of care in diabetes-2023. Diabetes Care.

[7] Nuche-Berenguer B, Moreno P, Esbrit P, Dapía S, Caeiro JR, Cancelas J, Haro-Mora JJ, Villanueva-Peñacarrillo ML (2009). Effect of GLP-1 treatment on bone turnover in normal, type 2 diabetic, and insulin-resistant states. Calcif Tissue Int.

[8] Cheng Y, Liu P, Xiang Q, Liang J, Chen H, Zhang H, Yang L (2022). Glucagon-like peptide-1 attenuates diabetes-associated osteoporosis in ZDF rat, possibly through the RAGE pathway. BMC Musculoskelet Disord.

[9] Viggers R, Rasmussen NH, Vestergaard P (2023). Effects of Incretin Therapy on Skeletal Health in Type 2 Diabetes-A Systematic Review. JBMR Plus.

[10] Eminov E, Hortu I, Akman L, Erbas O, Yavasoglu A, Cirpan T (2018). Exenatide preserves trabecular bone microarchitecture in experimental ovariectomized rat model. Arch Gynecol Obstet.

[11] Zhou Y, Xue X, Guo Y, Liu H, Hou Z, Chen Z, Wang N, Li F, Wang Y (2021). A quinoxaline-based compound ameliorates bone loss in ovariectomized mice. Exp Biol Med (Maywood).

[12] Conversano F, Franchini R, Greco A, Soloperto G, Chiriacò F, Casciaro E, Aventaggiato M, Renna MD, Pisani P, Di Paola M, Grimaldi A, Quarta L, Quarta E, Muratore M, Laugier P, Casciaro S (2015). A novel ultrasound methodology for estimating spine mineral density. Ultrasound Med Biol.

[13] Di Paola M, Gatti D, Viapiana O, Cianferotti L, Cavalli L, Caffarelli C, Conversano F, Quarta E, Pisani P, Girasole G, Giusti A, Manfredini M, Arioli G, Matucci-Cerinic M, Bianchi G, Nuti R, Gonnelli S, Brandi ML, Muratore M, Rossini M (2019). Radiofrequency echographic multispectrometry compared with dual X-ray absorptiometry for osteoporosis diagnosis on lumbar spine and femoral neck. Osteoporos Int.

[14] Caffarelli C, Tomai Pitinca MD, Al Refaie A, Ceccarelli E, Gonnelli S (2022). Ability of radiofrequency echographic multispectrometry to identify osteoporosis status in elderly women with type 2 diabetes. Aging Clin Exp Res.

[15] Starup-Linde J, Eriksen SA, Lykkeboe S, Handberg A, Vestergaard P (2014). Biochemical markers of bone turnover in diabetes patients-a meta-analysis, and a methodological study on the effects of glucose on bone markers. Osteoporos Int.

[16] Eller-Vainicher C, Cairoli E, Grassi G, Grassi F, Catalano A, Merlotti D, Falchetti A, Gaudio A, Chiodini I, Gennari L (2020). Pathophysiology and Management of Type 2 diabetes mellitus bone fragility. J Diabetes Res.

[17] Wu B, Fu Z, Wang X, Zhou P, Yang Q, Jiang Y, Zhu D (2022). A narrative review of diabetic bone disease: characteristics, pathogenesis, and treatment. Front Endocrinol (Lausanne).

[18] Hygum K, Starup-Linde J, Harsløf T, Vestergaard P, Langdahl BL (2017). Mechanisms in endocrinology: diabetes mellitus, a state of low bone turnover - a systematic review and meta-analysis. Eur J Endocrinol.

[19] Meng J, Ma X, Wang N, Jia M, Bi L, Wang Y, Li M, Zhang H, Xue X, Hou Z, Zhou Y, Yu Z, He G, Luo X (2016). Activation of GLP-1 receptor promotes bone marrow stromal cell osteogenic differentiation through β-catenin. Stem Cell Reports.

[20] Iepsen EW, Lundgren JR, Hartmann B, Pedersen O, Hansen T, Jørgensen NR, Jensen JE, Holst JJ, Madsbad S, Torekov SS (2015). GLP-1 receptor agonist treatment increases bone formation and prevents bone loss in weight-reduced obese women. J Clin Endocrinol Metab.

[21] Hygum K, Harsløf T, Jørgensen NR, Rungby J, Pedersen SB, Langdahl BL (2020). Bone resorption is unchanged by liraglutide in type 2 diabetes patients: a randomised controlled trial. Bone.

[22] Cai TT, Li HQ, Jiang LL, Wang HY, Luo MH, Su XF, Ma JH (2021). Effects of GLP-1 receptor agonists on bone mineral density in patients with type 2 diabetes mellitus: a 52-week clinical study. Biomed Res Int.

[23] Huang CF, Mao TY, Hwang SJ (2023). The effects of switching from dipep tidyl Peptidase-4 inhibitors to glucagon-like Peptide-1 receptor agonists on bone mineral density in diabetic patients. Diabetes Metab Syndr Obes.

[24] Zibellini J, Seimon RV, Lee CM, Gibson AA, Hsu MS, Shapses SA, Nguyen TV, Sainsbury A (2015). Does diet-induced weight loss lead to bone loss in overweight or obese adults? A systematic review and meta-analysis of clinical trials. J Bone Miner Res.

[25] Herrou J, Mabilleau G, Lecerf JM, Thomas T, Biver E, Paccou J (2024). Narrative review of effects of glucagon-like peptide-1 receptor agonists on bone health in people living with obesity. Calcif Tissue Int.

[26] Zhang YS, Zheng YD, Yuan Y, Chen SC, Xie BC (2021). Effects of anti-diabetic drugs on fracture risk: a systematic review and net work meta-analysis. Front Endocrinol (Lausanne).

[27] Trandafir AI, Sima OC, Gheorghe AM, Ciuche A, Cucu AP, Nistor C, Carsote M (2023). Trabecular bone score (TBS) in individuals with type 2 diabetes mellitus: an updated review. J Clin Med.

[28] Armamento-Villareal R, Sadler C, Napoli N, Shah K, Chode S, Sinacore DR, Qualls C, Villareal DT (2020). Weight loss in obese older adults increases serum sclerostin and impairs hip geometry but both are prevented by exercise training. J Bone Miner Res.

[29] Yang J, Park O-J, Kim J, Han S, Yang Y, Yun C-H, Han SH (2019). Adiponectin deficiency triggers bone loss by up-regulation of osteoclastogenesis and down-regulation of osteoblastogenesis. Front Endocrinol.

[30] Simental-Mendía LE, Sánchez-García A, Linden-Torres E, Simental-Mendía M (2021). Impact of glucagon-like peptide-1 receptor agonists on adiponectin concentrations: a meta-analysis of randomized controlled trials. Br J Clin Pharmacol.

[31] Biver E, Salliot C, Combescure C, Gossec L, Hardouin P, Legroux-Gerot I, Cortet B (2011). Influence of adipokines and ghrelin on bone mineral density and fracture risk: a systematic review and meta-analysis. J Clin Endocrinol Metab.

[32] Register TC, Divers J, Bowden DW, Carr JJ, Lenchik L, Wagenknecht LE, Hightower RC, Xu J, Smith SC, Hruska KA, Langefeld CD, Freedman BI (2013). Relationships between serum adiponectin and bone density, adiposity and calcified atherosclerotic plaque in the African American-Diabetes Heart Study. J Clin Endocrinol Metab.

[33] Johansson H, Odén A, Lerner UH, Jutberger H, Lorentzon M, Barrett-Connor E, Karlsson MK, Ljunggren O, Smith U, McCloskey E, Kanis JA, Ohlsson C, Mellström D (2012). High serum adiponectin predicts incident fractures in elderly men: Osteoporotic fractures in men (MrOS) Sweden. J Bone Miner Res.

[34] Zhi X, Chen Q, Song S, Gu Z, Wei W, Chen H, Chen X, Weng W, Zhou Q, Cui J, Cao L (2020). Myostatin promotes osteoclastogenesis by regulating Ccdc50 gene expression and RANKL-Induced NF-κB and MAPK Pathways. Front Pharmacol.

[35] Reid IR, Baldock PA, Cornish J (2018). Effects of leptin on the skeleton. Endocr Rev.

[36] Deepika F, Bathina S, Armamento-Villareal R (2023). Novel adipokines and their role in bone metabolism: a narrative review. Biomedicines.

