# Supplementary material for: Glucagon-like Peptide-1 Receptor Agonists and Diabetic Osteopathy: Another Positive Effect of Incretines? A 12 Months Longitudinal Study
Source: Calcif Tissue Int. 2024 Jun 12;115(2):160–8. doi: 10.1007/s00223-024-01240-1 (PMC11246279; doi:10.1007/s00223-024-01240-1)
Supplement: Supplementary file 1 — Supplementary file1 (DOCX 17 KB) [file 223_2024_1240_MOESM1_ESM.docx]

Table 2 - Demographic, clinical and laboratory characteristics of the study population at baseline and after 12 months of therapy with the GLP-1RAs **semaglutide**

|  | **Baseline (N=29)** | **12 months (N=24)** | **p** |
| --- | --- | --- | --- |
| Age (yrs) | 66.18 ± 6.53 | 67.35 ± 6.25 | n.s. |
| Weight (Kg) | 80.96 ± 11.51 | 77.58 ± 11.02 | 0.01 |
| Height (cm) | 165.43 ± 6.27 | 165.02 ± 5.87 | n.s. |
| BMI (Kg/m^2^) | 29.67 ± 4.55 | 28.36 ± 4.37 | 0.01 |
| 25OHD (ng/ml) | 21.70 ± 6.80 | 22.41 ± 6.34 | n.s. |
| PTH (pg/ml) | 27.20 ± 21.92 | 29.12 ± 18.41 | n.s. |
| B-ALP (µg/L) | 12.92 ± 3.26 | 15.18 ± 2.94 | 0.05 |
| CTX (ng/L) | 0.178 ± 0.093 | 0.218 ± 0.094 | 0.05 |
| Sclerostin (pmol/L) | 51.62 ± 26.93 | 53.80 ± 6.06 | n.s. |
| Adiponectin (µg/ml) | 9.65 ± 2.94 | 12.78 ± 3.18 | 0.01 |
| Myostatin (ng/ml) | 12.85 ± 3.36 | 11.15 ± 2.65 | 0.05 |
| DXA LS-BMD (g/cm^2^) | 1.152 ± 0.173 | 1.133 ± 0.163 | 0.05 |
| DXA TH-BMD (g/cm^2^) | 1.051 ± 0.126 | 1.027 ± 0.160 | 0.05 |
| DXA FN-BMD (g/cm^2^) | 0.839 ± 0.114 | 0.778 ± 0.119 | 0.05 |
| REMS LS-BMD (g/cm^2^) | 0.907 ± 0.086 | 0.881 ± 0.078 | 0.05 |
| REMS TH-BMD (g/cm^2^) | 0.907 ± 0.145 | 0.864 ± 0.134 | 0.05 |
| REMS FN-BMD (g/cm^2^) | 0.759 ± 0.106 | 0.719 ± 0.106 | 0.05 |
|  |  |  |  |

Table 3 - Demographic, clinical and laboratory characteristics of the study population at baseline and after 12 months of therapy with the GLP-1RAs **dulaglutide**

|  | **Baseline (N=36)** | **12 months (N=30)** | **p** |
| --- | --- | --- | --- |
| Age (yrs) | 66.68 ± 9.97 | 67.39 ± 8.56 | n.s. |
| Weight (Kg) | 86.07 ± 13.52 | 82.43 ± 11.49 | 0.01 |
| Height (cm) | 170.00 ± 9.29 | 169.63 ± 8.95 | n.s. |
| BMI (Kg/m^2^) | 30.69 ± 4.57 | 29.25 ± 2.94 | 0.01 |
| 25OHD (ng/ml) | 20.85 ± 11.07 | 22.05 ± 8.73 | n.s. |
| PTH (pg/ml) | 28.58 ± 23.38 | 30.02 ± 23.48 | n.s. |
| B-ALP (µg/L) | 13.07 ± 3.51 | 15.58 ± 2.85 | 0.05 |
| CTX (ng/L) | 0.193 ± 0.092 | 0.241 ± 0.101 | 0.05 |
| Sclerostin (pmol/L) | 52.90 ± 29.26 | 54.18 ± 35.78 | n.s. |
| Adiponectin (µg/ml) | 9.34 ± 7.66 | 11.71 ± 8.72 | 0.01 |
| Myostatin (ng/ml) | 13.21 ± 2.47 | 11.28 ± 1.38 | 0.05 |
| DXA LS-BMD (g/cm^2^) | 1.225 ± 0.291 | 1.193 ± 0.258 | 0.05 |
| DXA TH-BMD (g/cm^2^) | 1.015 ± 0.177 | 0.983± 0.219 | 0.05 |
| DXA FN-BMD (g/cm^2^) | 0.926 ± 0.204 | 0.875 ± 0.19 8 | 0.05 |
| REMS LS-BMD (g/cm^2^) | 0.926 ± 0.111 | 0.901 ± 0.133 | n.s. |
| REMS TH-BMD (g/cm^2^) | 0.949 ± 0.149 | 0.907 ± 0.150 | 0.05 |
| REMS FN-BMD (g/cm^2^) | 0.799 ± 0.135 | 0.783 ± 0.106 | n.s. |
